# 2106. Desirability of Outcome Ranking (DOOR): Application to a Phase 3 Registrational Trial Evaluating Sulopenem for Patients with Complicated Intra-abdominal Infection (cIAI)

**DOI:** 10.1093/ofid/ofad500.1730

**Published:** 2023-11-27

**Authors:** Steven I Aronin, Michael W Dunne, Jayanti Gupta, Sailaja Puttagunta

**Affiliations:** Iterum Therapeutics, Old Saybrook, Connecticut; Iterum Therapeutics, Old Saybrook, Connecticut; Iterum Therapeutics, Old Saybrook, Connecticut; Iterum Therapeutics, Old Saybrook, Connecticut

## Abstract

**Background:**

The DOOR approach has been proposed as an improved way to evaluate novel anti-infective agents by focusing on benefits and harms and providing an assessment of the patient experience. We conducted a Phase 3 cIAI trial comparing IV ertapenem (stepped down to either oral ciprofloxacin and metronidazole or amoxicillin-clavulanate) to IV sulopenem (stepped down to oral sulopenem etzadroxil/probenecid). Using the FDA’s current definition of a successful response (clinical response at Day 28 / Test-Of-Cure (TOC) in the microbiological intent-to-treat (micro-ITT) population using a non-inferiority margin of 10%, sulopenem’s overall success rate was 85.5% while ertapenem’s was 90.2% (treatment difference -4.7%, 95% CI: -10.3, 1.0). In all other study populations including the intent-to-treat (ITT), modified ITT, clinically evaluable (CE), and microbiologically evaluable (ME) populations the lower limit of confidence interval was above -10.0. To further understand these trial results, an analysis using the DOOR methodology was performed post hoc.

**Table 1:**
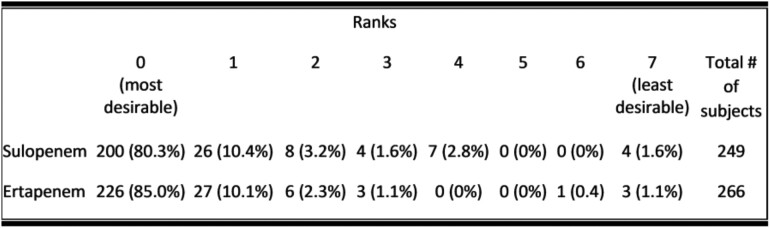
Desirability of Outcome Rankings by Treatment Arm

**Methods:**

The DOOR analysis strategy, developed by the Antibacterial Resistance Leadership group (ARLG), was retrospectively applied to our registrational drug trial for cIAI (SURE-3) to estimate the probability of a more desirable outcome for sulopenem.

**Results:**

The DOOR probability of a more desirable outcome is 47.4% [95% CI (44.1%, 50.8%)], indicating no significant difference between the sulopenem and ertapenem treatment arms for patients with cIAI. The probabilities for the analyses prioritizing efficacy and safety were identical to the original outcome ranking, and those for the individual components were very similar.

**Conclusion:**

Traditional endpoints used in registrational trials for cIAI may be inadequate. They evaluate safety and efficacy separately, and they fail to evaluate the cumulative impact of multiple clinical events. DOOR combines clinical efficacy and safety into a single endpoint that may be more reflective of an individual patient’s overall outcome. Applying DOOR to SURE-3 data showed no significant difference between the sulopenem and ertapenem treatment arms for patients with cIAI.

**Disclosures:**

**Steven I. Aronin, MD**, Iterum Therapeutics Limited: Stocks/Bonds **Michael W. Dunne, MD**, Iterum Therapeutics: Board Member|Iterum Therapeutics: Stocks/Bonds **Jayanti Gupta, PhD**, Iterum Therapeutics: Advisor/Consultant **Sailaja Puttagunta, MD**, Iterum Therapeutics Limited: Full time employee|Iterum Therapeutics Limited: Stocks/Bonds

